# AGR2 expression as a predictive biomarker for therapy response in esophageal squamous cell carcinoma

**DOI:** 10.1371/journal.pone.0276990

**Published:** 2022-11-03

**Authors:** Chih-Hung Lin, Han-Ni Chuang, Tzu-Hung Hsiao, V. Bharath Kumar, Chiung-Hung Hsu, Chih-Yang Huang, Li-Wen Lee, Chien-Lin Mao, Jiunn-Liang Ko, Chung-Ping Hsu

**Affiliations:** 1 Institute of Medicine, Chung Shan Medical University, Taichung, Taiwan; 2 Division of Thoracic Surgery, Department of Surgery, Taichung Veteran General Hospital, Taichung, Taiwan; 3 Department of Medical Research, Taichung Veterans General Hospital, Taichung, Taiwan; 4 Department of Public Health, Fu Jen Catholic University, New Taipei City, Taiwan; 5 Institute of Genomics and Bioinformatics, National Chung Hsing University, Taichung, Taiwan; 6 Department of Medical Laboratory Science and Biotechnology, Asia University, Taichung, Taiwan; 7 Cardiovascular and Mitochondrial Related Disease Research Center, Hualien Tzu Chi Hospital, Buddhist Tzu Chi Medical Foundation, Hualien, Taiwan; 8 Center of General Education, Buddhist Tzu Chi Medical Foundation, Tzu Chi University of Science and Technology, Hualien, Taiwan; 9 Department of Medical Research, China Medical University Hospital, China Medical University, Taichung, Taiwan; 10 Department of Medical Oncology and Chest Medicine, Chung Shan Medical University Hospital, Taichung, Taiwan; 11 Division of Thoracic Surgery, Department of Surgery, Buddhist Tzu Chi General Hospital, Hualien, Taiwan; Peter MacCallum Cancer Centre, AUSTRALIA

## Abstract

Despite multidisciplinary therapy, the prognosis is poor for esophageal squamous cell carcinoma (ESCC). In the locally advanced stage, neoadjuvant chemoradiotherapy (nCRT) followed by surgery could provide survival benefits to some patients. Here, we aimed to identify for tumor therapy response a biomarker based on RNA sequencing. We collected endoscopic biopsies of 32 ESCC patients, who were divided according to nCRT response, into two groups: the complete response group (n  =  13) and the non-complete response group (n  =  19). RNA-sequencing data showed that 464 genes were differentially expressed. Increased in non-complete response group, 4 genes increased expressions were *AGR2 (anterior gradient 2)*, *GADD45B (growth arrest and DNA damage inducible beta)*, *PPP1R15A (protein phosphatase 1 regulatory subunit 15A)* and *LRG1 (leucine rich alpha-2-glycoprotein 1)*. The areas under the curve (AUC) of the *AGR2* gene was 0.671 according to read counts of RNA-seq and therapy response of nCRT. *In vitro* study showed that apoptosis cell was significantly increased in the *AGR2-*knockdown TE-2 cell line treated with cisplatin and 5-Fluorouracil (5-FU), when compared with si-control. Results suggest that in ESCC, the *AGR2* gene is a promising and predictive gene marker for the response to anti-tumor therapy.

## Introduction

*Esophageal squamous cell carcinoma (ESCC*) is a very common human cancer, and ranked 6th most common cancer worldwide. In Taiwan, it is the 5th leading cause of death among men, particularly prevalent in South-East and Central Asia. Esophageal cancer is histologically classified as either squamous cell carcinoma or adenocarcinoma [1]. Surgical resection is the main-stream treatment for early-stage esophageal carcinoma. For locally advanced esophageal carcinoma. The approach of multidisciplinary therapy, like radiotherapy, chemotherapy, and surgery, has been developed to prolong the patient survival. Despite this, the prognosis remains poor [2, 3]. Previous studies reported that Neoadjuvant chemoradiotherapy (nCRT) followed by surgery is a common multidisciplinary treatment for resectable esophageal carcinoma [4–11]. But the prognosis remains disappointing, with >50% of patients showing poor response to nCRT [12–15]. No simple and reliable criterion is available currently to determine the success of such therapy. According to the clinicopathologic and gene-expression profiles, some studies reported that tumor size and molecular makers, such as ERCC1, GNAS T93C, ABCB1 C3435T, might be associated with the response of chemotherapy or radiotherapy [8, 16–20]. These studies are mainly based on the post-treatment specimen as treatment-naïve specimens before nCRT are not available. Therefore, it is difficult to apply in clinical practice. Here, we aim to identify biomarkers from treatment-naïve specimens that allow early prediction the response to nCRT. Results would be useful to develop alternative personalized therapy or targeted therapy based on biomarkers.

As broad tumor profiling becomes a common component of cancer care, next-generation sequencing (NGS) is increasingly used in many areas of cancer research and clinical settings. Furthermore, endoscopic biopsies are suitable for targeted NGS, which provides quality sequencing data and accurate information on mutations [21–23]. NGS is a tool that is also widely available to gastroenterologists [21–23]. In this study, we aimed to identify potential genes for predicting response to therapy based on NGS biopsy samples from ESCC patients. Based on function analysis results, we chose AGR2 to perform further investigation. Results showed that silencing AGR2 enhances sensitivity to the cytotoxic effects of cisplatin and 5-fluorouracil (5-FU). We concluded that AGR2 is a potential gene marker for predicting response to ESCC therapy.

## Materials and methods

### Patient selection

From January 1, 2016, to December 31, 2018, we retrospectively enrolled 32 ESCC patients who had undergone nCRT at the Taichung Veterans General Hospital. These patients each had one endoscopic specimen of pre-treatment biopsy (treatment-naïve tissue) and another specimen of post-treatment biopsy. Samples of surgically resected tumors after nCRT were obtained from the Biobank of Taichung Veterans General Hospital. We collected their clinical information such as age, sex, surgery type, complete or incomplete resection, histologic subtype, tumor stage, clinical image data, and therapeutic response. Both the data collection procedure and the gene expression analysis of tumor tissues were approved by the Institutional Review Board of Taichung Veterans General Hospital (IRB TCVGH No: CE17279A). All patients did not contain minors and other vulnerable groups provided written informed consent to participate in this study before enrollment.

### RNA sequencing and gene expression analysis

RNA libraries were generated using the TruSeq Stranded mRNA Library Prep Kit (Illumina, SanDiego, CA, USA) with 1μg of total RNA from all samples following the manufacturer’s instructions. The prepared library was sequenced with paired-end runs using the Illumina HiSeq 2500 sequencer. RNA reads were mapped onto the human reference genome GRCh37 using the HISAT2aligner tool [24]. Read counts were calculated using feature Counts [25] and gene expression profiles were identified using DESeq2 [26]. The DAVID functional tool (the Database for Annotation, Visualization, and Integrated Discovery, https://david.ncifcrf.gov/) was used for functional annotation of differentially expressed genes. The Metascape (http://metascape.org/gp/index.html#/main/step1) online tools were used to analyze Gene Ontology (GO) analysis and Kyoto Encyclopedia of Genes and Genomes (KEGG) pathway enrichment [27].

### Cell line and culture conditions

We used two esophageal cancer cell lines (CE48T/VGH, and CE146T/VGH). Each was cultured in Dulbecco’s modified Eagle’s medium (Gibco, Grand Island, NY, USA) supplemented with 2mM L-glutamine (Gibco, Grand Island, NY, USA), 10% fetal bovine serum (Gibco, Grand Island, NY, USA), 100 U/mL penicillin-streptomycin (Gibco, Grand Is-land, NY, USA), and 10mM non-essential amino acids (Gibco, Grand Island, NY, USA). TE-2 cells were cultured in the same medium supplemented with 1mM sodium pyruvate (Gib-co, Grand Island, NY, USA). All cells were cultured in a 5% CO2 atmosphere at 37°C.

### RNA interference (small interfering RNA) analysis

The RNA interference (RNAi) technology has revolutionized biological discovery, target discovery, and validation processes. A SmartPool of 4 siRNA sequences derived from the coding sequence of AGR2 and individual duplex and control siRNA were designed and purchased from Dharmacon (Lafayette, CO, USA). The following siRNAs were used: AGR2 siRNA no.1: 5′—GCUGAAGACUGAAUUGUAA-3′, no.2: 5’-GCAACAAACCCUUGAUGAU-3′, no.3: AGUCAAACCUGGAGCCAAA-3′, and no.4 5′-UGAAGAAAGCUCUCAAGUU-3′. The control siRNA was non-targeting pool sequences that included the following: no. 1: 5′-UGGUUUACAUGUCGACUAA-3′, no. 2: 5′-UGGUUUACAUGUUGUGUGA-3′, no. 3: 5′-UGGUUUACAUGUUUUCUGA-3′, and no. 4: 5′-UGGUUUACAUGUUUUCCUA-3′. Each freeze-dried siRNA was dissolved in RNase-free water.

Using siRNA, we knocked down AGR*2* gene expression in esophageal cancer cells. The *Trans*IT-X2 Dynamic Delivery System reagent (Mirus Bio, Madison, WI, USA) procedure was used to forward-transfect siRNA into the esophageal cancer cells. The esophageal cancer cell cells were put in 6-well culture plates at a density of 4.0–6.0 ×10^5^ cells/well, and cultured in 2 mL growth medium for 24 hr. Cells were transfected with siRNA to a final concentration of 25 nM as diluted with the *TransIT-X2* transfection reagent. Subsequently, cells were incubated with 5% CO_2_ at 37°C for 72 hr. Finally, cells were harvested and assayed for the knockdown of target gene expression.

### Reverse transcription and quantitative polymerase chain reaction

The total RNA was extracted using the AllPrep DNA/RNA Mini Kit, following the manufacturer’s protocol (cat. 80204). Reverse transcription was done using the SuperScript^TM^ IV Reverse Transcriptase protocol (Invitrogen, Vilnius, Lithuania). Quantitative reverse-transcription polymerase chain reaction was done using the FastStart TaqMan Probes system (Cat.4913947001, Roche Diagnostics, Indianapolis, IN, USA) with AGR2 specific primers. The analysis was performed on a StepOne Plus Real‐Time PCR System (Applied Biosystems, Foster, CA). Glyceraldehyde 3-phosphate dehydrogenase was used as endogenous control to quantify determine the relative expression levels of target genes using the2^-ΔΔct^ method.

### Reagents

Cisplatin (P4394) and 5-fluorouracil (F6627) were purchased from Sigma-Aldrich, ST Louis, MO. Cisplatin was dissolved in double-distilled water. 5-FU was dissolved in dimethyl sulfoxide (DMSO) (Sigma-Aldrich, ST Louis, MO). Same solvent was used in the control experiment.

### MTT assay

To determine the cytotoxicity of the combined effect of *AGR2* knockdown and chemotherapeutic agents, cells were first put in 24-well culture plates for 24 hr. Then, cells were transfected with siRNA. After 24 hr, we treated the cells with cisplatin (2.0–6.0μM) and 5-FU (3.0–20.0μM) for 72hr. Cell viability was evaluated using the MTT assay. The medium was removed and cells were washed twice with phosphate-buffer saline (PBS). Then, 500 μL MTT solutions (1mg/mL) (Biomatik, Ontario, Canada) were added, and preparations incubated at 37°C for 30 min. The MTT solution was removed and replaced with 200 μL DMSO. Subsequently, cells were incubated for 5 min. We transferred 100 μL DMSO of dissolved cells into 96-well enzyme-linked immunosorbent assay (ELISA) plates to measure absorbance at 570/670 nm using an ELISA reader. Each experimental data point represents the average value of three replicates.

### Annexin V/propidium iodide apoptosis assay

The Annexin V-FITC Apoptosis Detection Kit was purchased from BioVision. Annexin V and propidium iodide (PI) double staining was performed following the manufacturer’s instructions. After staining, cells were analyzed with the flow cytometry.

### Western blotting

The western blot was used to determine levels of AGR2 and associated proteins. Cells were first washed with PBS and lysed in RIPA lysis buffer (APOLO, Hsinchu, Taiwan) containing 50mM Tris, 0.15M NaCl, 1% NP-40, 0.5% sodium deoxycholate, and 0.1% SDS supplemented with a protease inhibitor cocktail (MCE, Monmouth Junction, NJ, USA). Protein concentrations were detected using a protein assay kit (Bio-Rad, Hercules, CA, USA). Equal amounts of proteins (30 μg) were subjected to sodium dodecyl sulfate 8% -12% polyacrylamide gel electrophoresis. Fractionated proteins were transferred to Hybond-P PVDF membranes (Millipore, Darmstadt, DE). Membranes were blocked with PBS containing 5% nonfat milk and 0.2% Tween 20. For the detection of human anti-AGR2 (Invitrogen, Vilnius, Lithuania) and anti-β-actin (Sigma-Aldrich, St Louis, MO), the membranes were incubated overnight at 4°C, followed by the addition of anti-mouse IgG or anti-rabbit IgG antibody linked to Horseradish peroxidase (Jackson, West Grave, PA, USA). Blots were finally developed using an enhanced chemiluminescence reagent (Millipore, Darmstadt, DE).

### Statistical analyses

Statistical analyses were performed using the paired two-way analysis of variance with Tukey’s test. All results reflect the mean ± standard error of the mean data obtained from at least three independent experiments. Statistical was set defined as *p* <0.05.

## Results

### Clinical characteristic

We collected 32 patients with ESCC. Their clinicopathological characteristics are summarized in Table 1. Standard protocols for patients with operable esophageal cancer are bridfed as follows. Chemotherapy is given concurrently with cisplatin 20 mg/ml iv for 1 hour and fluorouracil 800 mg/ml iv for 24 hours on a daily basis from day 1 to 4 (cycle1), and from day 29 to 32 (cycle 2) with radiotherapy. Radiotherapy is performed 5 days per week, with a daily dose of 180 Gy over a total course of 5 to 6 weeks. Surgery was performed 4 to 6 weeks after completing nCRT. The procedure included thoracoscopic esophagectomy, at least 2-eld lymph node dissection and esophagus reconstruction with gastric tube. Patients had an average age of 59.9 years (range 48 to 82). They were divided into two groups according to their response to nCRT; Complete response group (n  =  13) and non-complete response group (n  =  19) (Table 2). Four patients in the non-complete response group and two patients in the complete response group did not undergo surgery after nCRT. The response status of these patients was confirmed by clinical evaluation and endoscopic biopsy.

**Table 1:** Clinicopathological characteristics of 32 patients with esophageal cancer before neoadjuvant chemoradiotherapy.

			Total	Complete response	Non-complete response
**No. of patients (n)**			32	13	19
**Age (mean)**			48–82 (59.9)	48–73 (59.6)	48–82 (60.0)
**Gender**	Male		31	12	19
	Female		1	1	0
**T stage**		T1	1	1	0
		T2	3	1	2
		T3	27	11	16
		T4	1	0	1
**N stage**		N0	5	1	4
		N1	13	5	8
		N2	11	6	5
		N3	3	1	2
**M stage**		M0	31	12	19
		M1	1	1	0

**Table 2:** Clinicopathological characteristics of patients with esophageal cancer after neoadjuvant chemoradiotherapy.

		Total	Complete response	Non-complete response
**No. of patients (n)**		32	13	19
**T stage**	T0	11	11	0
	T1	5	0	5
	T2	4	0	4
	T3	5	0	5
	T4	1	0	1
**N stage**	N0	20	11	9
	N1	5	0	5
	N2	1	0	1
	N3	0	0	0
**M stage**	M0	26	11	15
	M1	0	0	0

### RNA expressions were different between complete response and non-complete response groups

**Fig 1:**
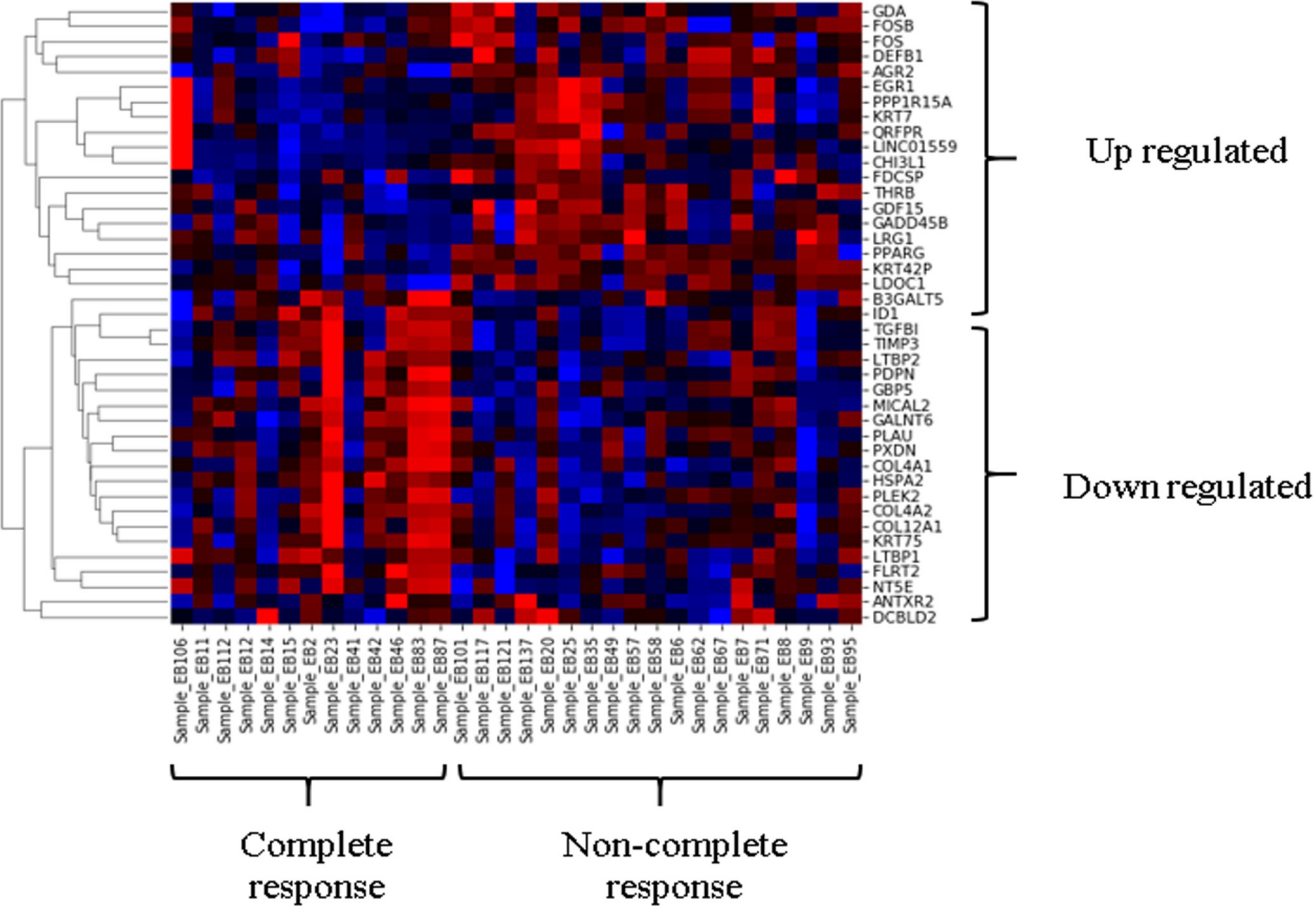


RNA sequencing reads were mapped against the human genome assembly (Ensembl Build 37) using TopHat (v2.1.1). We identified 464 differentially expressed genes (fold change >2 or <2, and a DESeq *p*-value of < 0.05). In non-complete group, 240 genes were up-regulated, and 224 genes were down-regulated. The fold changes of top 20 up-regulated genes and top 20 down-regulated genes are presented in Table 3 and S1 Table. Unsupervised hierarchical clustering of 20 up-regulated and 20 down-regulated genes also revealed differences in complete response or non-complete response of nCRT (Fig 1).

**Table 3:** Top 20 up-regulated expressed genes in ESCC according to RNA-sequence data.

Genes	Gene symbol	Base Mean	log2 Fold Change	lfcSE	stat	P-value
ENSG00000170345	FOS	20760.6	1.5	0.4	−2.9	3.20E-03
ENSG00000120738	EGFR1	10277.2	1.3	0.4	−2.8	5.10E-03
ENSG00000125968	ID1	6378.8	1.0	0.3	−3.6	3.60E-04
ENSG00000087074	PPP1R15A	6320.3	1.3	0.4	−2.6	9.80E-03
ENSG00000219507	FTHL8	3073.3	1.0	0.4	−2.8	4.80E-03
ENSG00000171236	LRG1	2813.5	1.1	0.4	−3.2	1.40E-03
ENSG00000106541	AGR2	2668.2	1.4	0.4	−2.9	3.90E-03
ENSG00000164825	DEFB1	2551.2	1.5	0.3	−3.0	2.80E-03
ENSG00000125740	FOSB	2461.1	1.6	0.3	3.3	8.10E-04
ENSG00000135480	KRT7	1706.7	1.3	0.4	−2.9	3.70E-03
ENSG00000099860	GADD45B	1513.3	1.1	0.4	3.2	1.50E-03
ENSG00000133048	CHI3L1	1011.0	1.2	0.4	3.4	7.10E-04
ENSG00000162896	PIGR	391.5	1.4	0.4	−3.5	4.20E-04
ENSG00000180861	LINC01559	376.6	1.3	0.4	−3.0	3.00E-03
ENSG00000182195	LDOC1	336.0	1.0	0.4	3.3	1.20E-03
ENSG00000119125	GDA	298.6	1.8	0.4	3.8	1.50E-04
ENSG00000151090	THRB	241.5	1.2	0.4	3.5	4.80E-04
ENSG00000214514	KRT42P	238.7	1	0.4	−4.6	3.40E-06
ENSG00000132170	PPARG	217.1	1.1	0.4	−3.0	2.80E-04
ENSG00000181617	FDCSP	210.1	1.2	0.4	3.5	4.60E-04

**Fig 2:**
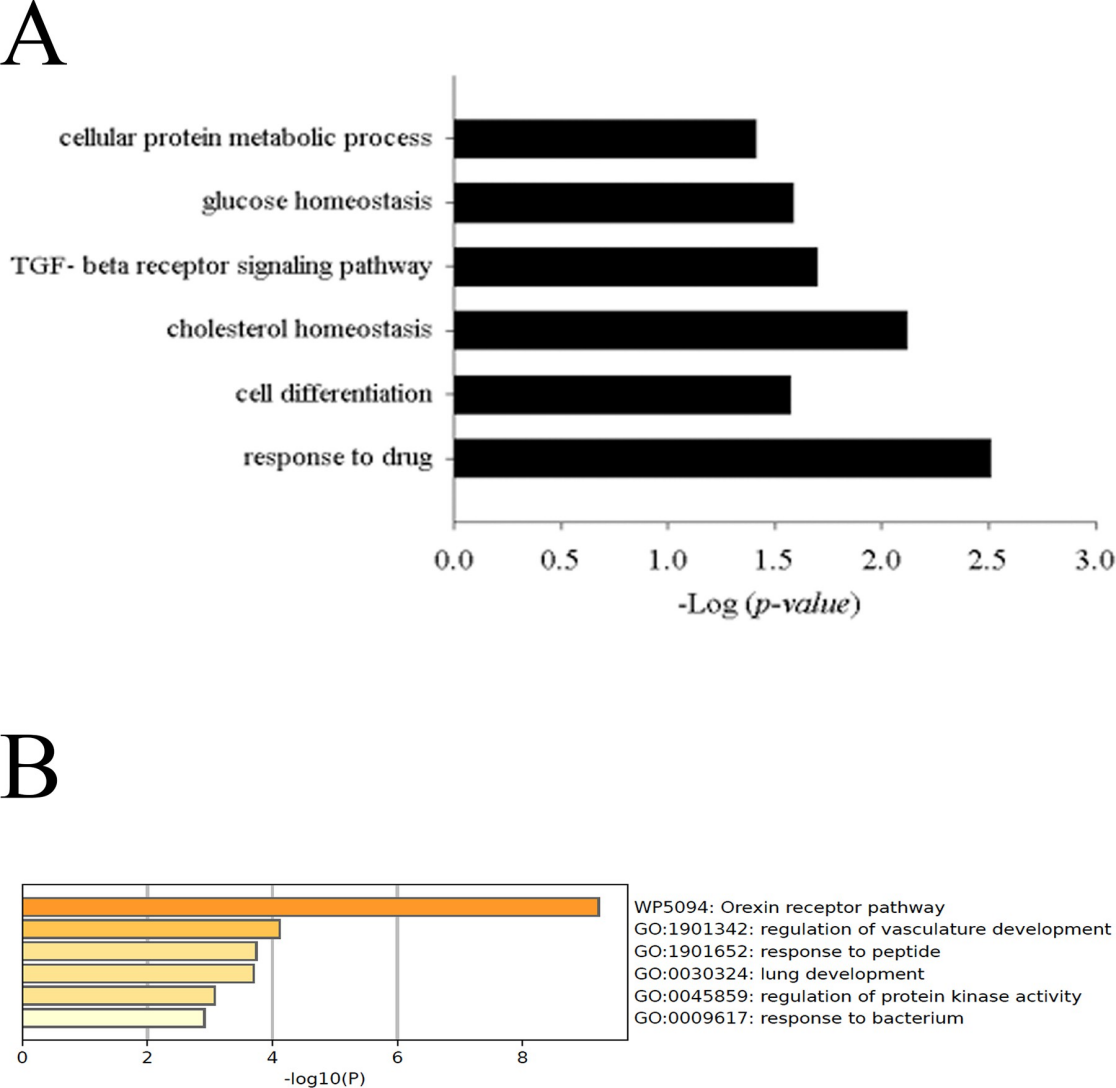


To explore potential functions of the differentially expressed genes and their controlled biological processes, we used the Database for Annotation, Visualization, and Integrated Discovery (DAVID) [28, 29]. Up-regulated genes in esophageal cancer with non-complete response were grouped 60 clusters, among which 30 clusters had *P-value*s <0.05. These genes are associated with the cellular protein metabolism process, glucose homeostasis, TGF-beta receptor signal response pathway, cholesterol homeostasis, cell differentiation and response to the drug (Fig 2A and S2 Table). The results of the analysis using Metascape are shown in Fig 2B. The up-regulated gene were mainly associated with 6 GO Biological Processes, including Orexin receptor pathway, regulation of vasculature development, response to peptide, lung development, regulation of protein kinase activity and response to bacterium. In addition, we analyzed down-regulated genes in esophageal cancer with non-complete response (S2 Fig). These genes are associated with extracellular matrix disassembly, collagen catabolic process, angiogenesis, immune response, cell adhesion and extracellular matrix organization (S2A Fig). The down-regulated gene were mainly associated with 9 GO Biological Processes, including NABA core matrisome, KRAS.DF.V1 up, PID integrin3 pathway, NABA ECM Glycoproteins, supramolecular fiber organization, BMI1 DN MEL18 DN.V1 up, wound heading, morphogenesis of an epithelium and positive regulation of cellular component biogenesis (S2B Fig).

**Fig 3:**
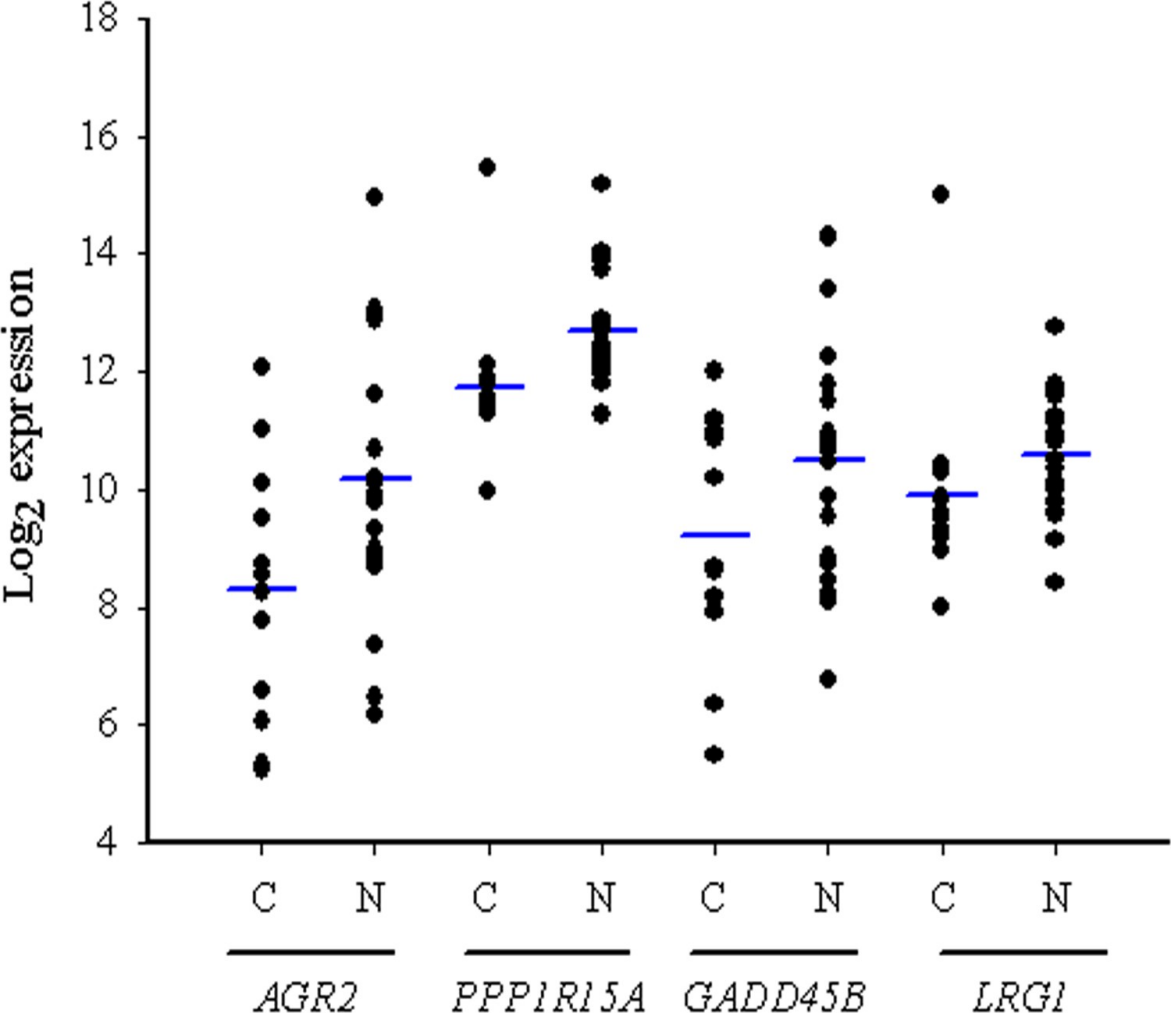


**Fig 4:**
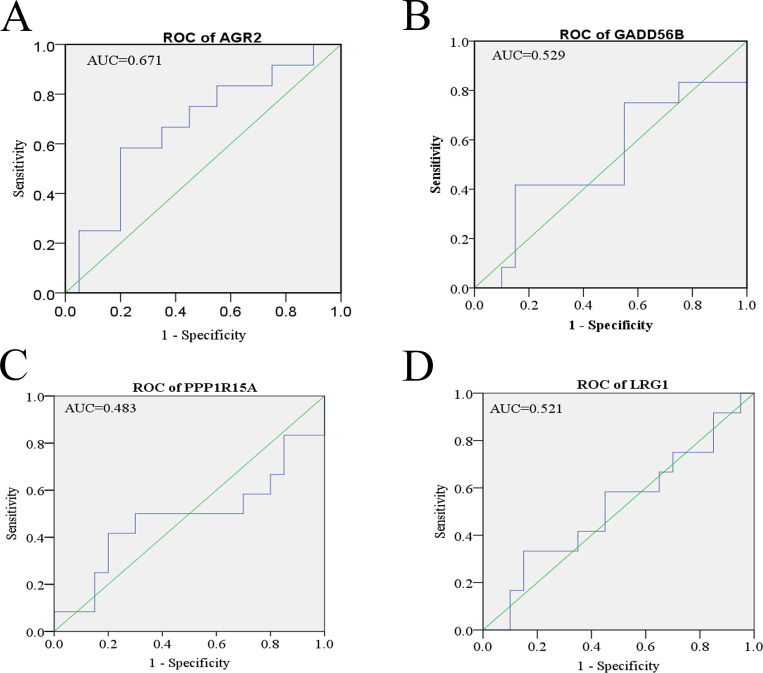


We first compared between complete response and non-complete response groups, expressions of up-regulated genes. We then selected 4 genes that are associated with cell proliferation and cell migration: namely, *AGR2* [30], *PPP1R15A* [31], *GADD45B* [32], and *LRG1* [33]. RNA sequencing indicated that the 4 genes were significantly up-regulated in patients with non-complete response to nCRT compared with those with complete response (Fig 3). To determine their possible risks for therapy progression in esophageal cancer patients, we plotted receiver operating characteristic curves (ROC) using read counts of RNA-Seq and therapy response of nCRT. Notably, the areas under the curves (AUCs) of *AGR2*, *GADD45B*, *PPP1R15A* and *LRG1* genes were 0.671, 0.529, 0.483 and 0.521, respectively (Fig 4). Results suggest that AGR2 gene was associated with therapy response of nCRT in esophageal cancer patients.

### Knockdown of AGR2 in esophageal cancer cells were more sensitive to the cytotoxicity effect of cisplatin and 5-fluorouracil

**Fig 5:**
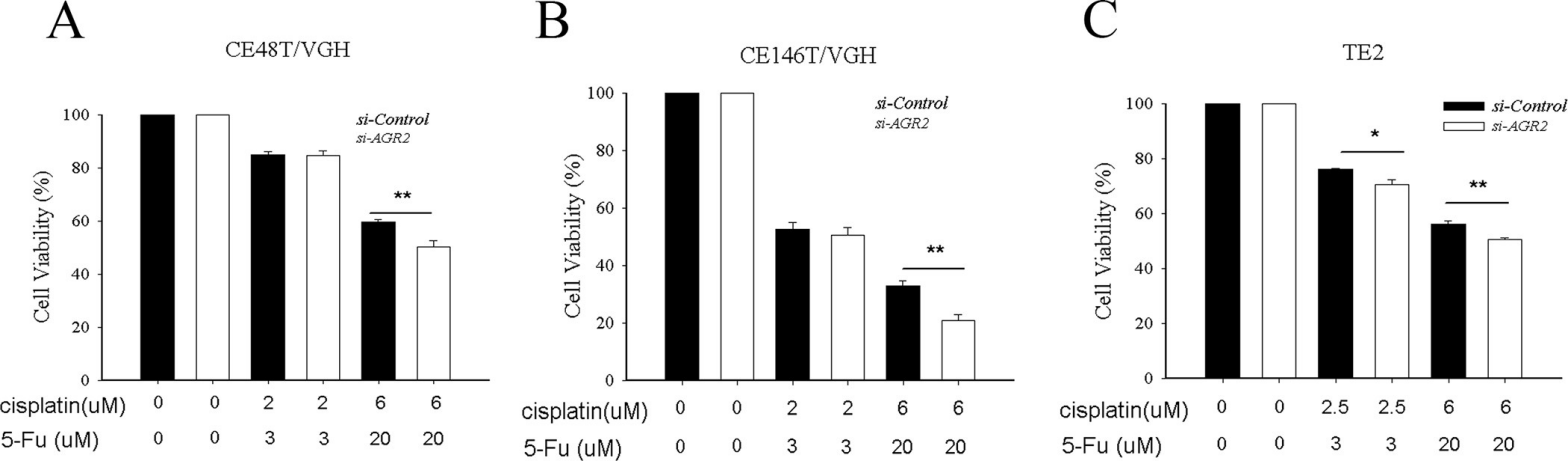


Previous studies reported that AGR2 is involved in head and neck squamous cell carcinoma by regulating cell transformation and epithelial-mesenchymal transition (EMT) signaling pathways [34], and that it also promotes tumor growth in esophageal adenocarcinoma [35]. In this study, we found that the *AGR2* mRNA p-regulated in patients with non-complete response before nCRT. Therefore, we selected AGR2 for further investigation. We applied the siRNA approach to knockdown AGR2 expression in cell lines of esophageal cancer (CE146T/VGH, TE2, and CE48T/VGH) and then performed the MTT assay. Western blot analysis showed that protein levels of AGR2 were significantly reduced in CE146T/VGH, TE2, and CE48T/VGH cells transfected with si-AGR2. (S1 Fig). MTT assay showed a lower cell viability of the *AGR2*-knockdown esophageal cell line following treatment with 2.5 μM cisplatin and 3 μM 5-FU, while those cell viabilities of CE48T/VGH and CE146T/VGH remained unchanged (Fig 5). Treatment with 6 μM cisplatin and 20 μM 5-FU on *AGR2*-knockdown cells (CE48T/VGH, CE146T/VGH and TE-2) led to a lower cell viability relative to the siRNA-control (Fig 5). These finding indicated that *AGR2* down-regulated cells were more sensitive to cisplatin and 5-FU combined treatment.

### Cisplatin and 5-FU induce apoptosis in AGR2-knockdown esophageal cancer cells

**Fig 6:**
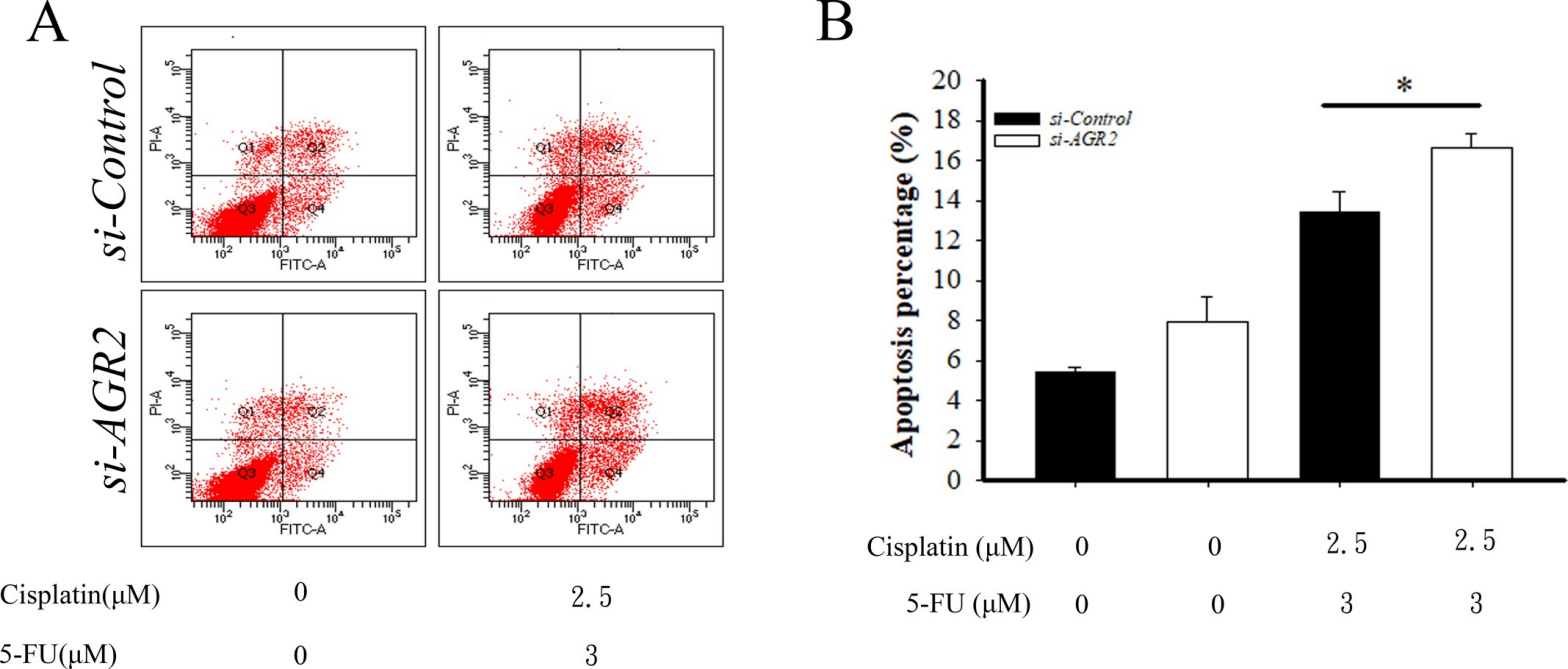


To determine if AGR2 modulates the sensitivity of esophageal cells to cisplatin and 5-FU, knockdown of *AGR2* in TE2 cells were incubated with 2.5 μM cisplatin and 3 μM 5-FU for 72hr. We then assessed *in vitro* effects on apoptosis induction using the Annexin V (Fig 6). We found on change in the percentage of apoptosis cells in the si-*AGR2* compared with si-control (5.4 ± 0.2% vs. 8.0 ± 1.3%). Of particular note, joint application of cisplatin and 5-FU on si-*ARG2* TE2 cells induced more apoptosis compared with the si-control (13.47± 1.3% vs. 16.6± 0.7%) (Fig 6). Results suggested that the cytotoxicity sensitivity to cisplatin and 5-FU in esophageal cancer was associated with AGR2 expression.

## Discussion

In this study, we have identified a biomarker to predict nCRT responses in esophageal cancer. Even though our specimens in ESCC patients were tiny and obtained only through gastroscopic biopsy before nCRT, we had found 464 differentially expressed genes that were associated with the response to nCRT. Among them, we further found that the expression of the anterior gradient gene, AGR2, was associated with the cytotoxicity of drug response from experiments on esophageal cell lines.

Anterior gradient genes were first found in *Xenopus laevis*. In humans, anterior gradient proteins are distributed mostly in endoderm-derived organs, such as the lungs, stomach, small intestine, colon, and prostate [30, 36]. AGR2 was initially found in human breast cancer specimens [30], and it is a member of the disulfide isomerase family of endoplasmic reticulum (ER) proteins that catalyze protein folding and thiol-disulfide interchange reactions [37]. Derepression of AGR2 was not only found in breast cancer cells [30, 38], but also in other common adenocarcinomas, including those derived from the esophagus [39], stomach [40], lungs [41], pancreas [42], ovaries [43], and prostate [44].

AGR2 in both breast cancer and prostate cancer is likely may be associated with endocrine status and treatment response. As an ER-localized molecular chaperone, AGR2 regulates the folding, trafficking, and assembly of cysteine-rich transmembrane receptors and the cysteine-rich intestinal glycoprotein mucin [45]. In the prostate carcinoma, AGR2 is induced by androgens [46].

In terms of protein function, AGR2 is involved in cell migration, and cellular transformation, and metastasis as well as being a p53 inhibitor [37, 45, 47]. The role of AGR2 has been implicated in inflammatory bowel disease and cancer progression [48]. Pohler et al., [49] reported that AGR2 promotes colony formation in lung cancer cells (H1299), while overexpressing AGR2 in undamaged cells does not change their cell-cycle parameters. Furthermore, in prostate cancer, extracellular AGR2 combines with vascular endothelial growth factor (VEGF), before activating VEGF receptor signalling and inducing angiogenesis. Intracellular AGR2 induces EMT gene transcription through stabilizing p65, and then facilitates metastatic processes [50]. Lucia et al., [51] showed that the function of AGR2 is reduced by TGF-β and maintains the epithelial phenotype by preventing the activation of key factors involved in the process of EMT in breast cancer. The Orexin receptor type 1 (Ox1R) had pro-apoptotic properties in esophageal cancer [52].

The condition of Barrett’s esophagus is known to precede esophageal adenocarcinoma. AGR2 is universally overexpressed in the epithelium of Barrett’s esophagus and esophageal adenocarcinoma [39, 49]. In esophageal adenocarcinoma, AGR2 expression also promotes tumor growth, cell migration, and cellular transformation [35]. Dong et al., further demonstrated that AGR2 in esophageal adenocarcinoma promotes tumor growth by inducing AGR2 expression of and regulates the Hippo signaling pathway co-activator [53].

Most researchers focused their studies on the relationship between AGRs and adenocarcinoma. However, very few of them considered AGR2 roles in squamous cell carcinoma. Ma et al., [34] reported that in head and neck squamous cell carcinoma, AGR2 expression is associated with tumor grade and tumor size. They also showed that radiotherapy and chemotherapy likely induce AGR2 expression. AGR2 expression may function as a survival factor and is remarkably associated with survivin, cyclin D1, ALDH1, Sox2, Oct4, and Slung. AGR2 may affect cell apoptosis, invasion, proliferation, metastasis, and the EMT signaling pathway in squamous cell carcinoma [34, 54, 55].

Although AGR2 levels correlate with nCRT response in ESCC, the underlying mechanism remain unclear. The p53 tumor suppressor gene is involved in the regulation of the cell cycle, apoptosis, and DNA repair. However, p53 has a high mutation frequency and its exact role on the prognosis of esophageal carcinoma remains debatable [56]. On the other hand, p21, which is a cell cycle regulator appears to be a more reliable marker for predicting nCRT responses in esophageal carcinoma. Furthermore, p21 is involved in multiple pathways that are independent of p53 [56, 57]. Therefore, we postulate a possible relationship between AGR2 and p21 expressions in ESCC.

AGR2 expression is known to be linked with drug resistance. In breast carcinoma, AGR2 expression in ER a-positive patients is associated with drug resistance to tamoxifen [58]. In lung cancer, AGR2 can modulate EGFR-TKI resistance in EGFR-mutant non-small cell carcinoma [59]. In prostate adenocarcinoma, AGR2 could enhance the antitumor effect of bevacizumab [50]. In pancreatic carcinoma, AGR2 expression is related to the response to gemcitabine [60], and in an animal model, blocking monoclonal antibodies against AGR2 and C4.4A resulted in the regression of tumor invasion and increased survival [61]. Therefore, the suppression of AGR2 may be a therapeutic option.

DiMaio et al., retrospectively examined 116 specimens of esophageal carcinoma. They demonstrated that the presence of diffuse AGR2 expression is highly sensitive to esophageal adenocarcinoma. However, focal expression of AGR2 was found only in 1/3 (36.59%) of ESCC specimens [62]. Because AGR2 is not universally expressed in ESCC, a predictor is of greater importance in esophageal adenocarcinoma. Valladares-Ayerbesand et al., AGR2 as a suitable candidate gene for the detection of circulating tumor cells in patients with gastrointestinal cancer, a finding that extends the clinical application of AGR2 [63].

## Supporting information

S1 Table Top 20 down-regulated expressed genes between complete response and non-complete response groups by RNA-seq. (DOCX)

S2 Table Functional analysis of Up-regulated genes. (DOCX)

S1 Fig Protein levels of AGR2 decreased in esophageal cancer cell lines with AGR2-knockdown. The amounts of 25μM control siRNA (si-Control) and 25μM AGR2 siRNA (si-AGR2) were transfected into esophageal cells. The loading control was β-actin expression level. (A) CE146T/VGH, (B) TE-2 and CE48T/VGH.
(TIF)

S2 Fig The bar graph showed the down-regulated gene expression pattern according to functional enrichment analysis from DAVID (A) and Metascape (B) online.
(TIF)
