# Supplementary material for: AGR2 expression as a predictive biomarker for therapy response in esophageal squamous cell carcinoma
Source: PLoS One. 2022 Nov 3;17(11):e0276990. doi: 10.1371/journal.pone.0276990 (PMC9632826; doi:10.1371/journal.pone.0276990)
Supplement: S1 Table — (DOCX) [file pone.0276990.s001.docx]

**S1 Table. Top 20 down-regulated expressed genes between complete response and non-complete response groups by RNA-seq.**

| **Genes** | **Gene_symbol** | **BaseMean** | **Log2FoldChange** | **lfcSE** | **Stat** | **Pvalue** |
| --- | --- | --- | --- | --- | --- | --- |
| ENSG00000120708 | TGFBI | 11521.08 | -1.003005465 | 0.356195 | -2.81589 | 4.86E-03 |
| ENSG00000100234 | TIMP3 | 1842.126 | -1.00693219 | 0.385138 | -2.61447 | 8.94E-03 |
| ENSG00000119681 | LTBP2 | 1073.812 | -1.024458994 | 0.308252 | -3.32344 | 8.89E-04 |
| ENSG00000162493 | PDPN | 2764.441 | -1.024595256 | 0.341998 | -2.99591 | 2.74E-03 |
| ENSG00000154451 | GBP5 | 1606.787 | -1.027334204 | 0.401958 | -2.55582 | 1.06E-02 |
| ENSG00000133816 | MICAL2 | 892.8879 | -1.032556717 | 0.316219 | -3.26533 | 1.09E-03 |
| ENSG00000139629 | GALNT6 | 1371.151 | -1.051572834 | 0.339178 | -3.10036 | 1.93E-03 |
| ENSG00000122861 | PLAU | 8795.881 | -1.057976444 | 0.353883 | -2.98962 | 2.79E-03 |
| ENSG00000130508 | PXDN | 1588.615 | -1.095806051 | 0.397797 | -2.75469 | 5.87E-03 |
| ENSG00000187498 | COL4A1 | 5106.233 | -1.102783746 | 0.375702 | -2.93526 | 3.33E-03 |
| ENSG00000126803 | HSPA2 | 2213.342 | -1.106328534 | 0.348795 | -3.17185 | 1.51E-03 |
| ENSG00000100558 | PLEK2 | 2207.103 | -1.117106543 | 0.347849 | -3.21147 | 1.32E-03 |
| ENSG00000134871 | COL4A2 | 5094.92 | -1.125377225 | 0.378869 | -2.97036 | 2.97E-03 |
| ENSG00000111799 | COL12A1 | 5486.863 | -1.14674255 | 0.399193 | -2.87265 | 4.07E-03 |
| ENSG00000170454 | KRT75 | 1003.966 | -1.25044003 | 0.402345 | -3.10788 | 1.88E-03 |
| ENSG00000049323 | LTBP1 | 3801.779 | -1.316450594 | 0.343064 | -3.83734 | 1.24E-04 |
| ENSG00000185070 | FLRT2 | 1153.484 | -1.342663913 | 0.409922 | -3.27541 | 1.06E-03 |
| ENSG00000135318 | NT5E | 1298.565 | -1.400462079 | 0.394201 | -3.55266 | 3.81E-04 |
| ENSG00000163297 | ANTXR2 | 982.8767 | -1.436756733 | 0.388166 | -3.7014 | 2.14E-04 |
| ENSG00000057019 | DCBLD2 | 1534.859 | -1.497487002 | 0.300548 | -4.98252 | 6.28E-07 |
