# Supplementary material for: AGR2 expression as a predictive biomarker for therapy response in esophageal squamous cell carcinoma
Source: PLoS One. 2022 Nov 3;17(11):e0276990. doi: 10.1371/journal.pone.0276990 (PMC9632826; doi:10.1371/journal.pone.0276990)
Supplement: S2 Table — (DOCX) [file pone.0276990.s002.docx]

**S2** **Table. Functional analysis of Up-regulated genes.**

| Term | Count | | P-Value |
| --- | --- | --- | --- |
| nervous system development | 9 | 4.70E-04 | |
| neuronal action potential | 4 | 7.10E-04 | |
| membrane depolarization during action potential | 4 | 7.10E-04 | |
| response to drug | 8 | 3.10E-03 | |
| cholesterol transport | 3 | 4.50E-03 | |
| cholesterol homeostasis | 4 | 7.70E-03 | |
| regulation of postsynaptic membrane potential | 3 | 8.40E-03 | |
| cholesterol efflux | 3 | 1.10E-02 | |
| triglyceride catabolic process | 3 | 1.10E-02 | |
| membrane depolarization | 3 | 1.10E-02 | |
| sodium ion transport | 4 | 1.50E-02 | |
| chemical synaptic transmission | 6 | 1.80E-02 | |
| detection of glucose | 2 | 1.90E-02 | |
| transforming growth factor beta receptor signaling pathway | 4 | 2.00E-02 | |
| negative regulation of lipase activity | 2 | 2.50E-02 | |
| glucose homeostasis | 4 | 2.60E-02 | |
| cell differentiation | 8 | 2.70E-02 | |
| receptor-mediated endocytosis | 5 | 3.00E-02 | |
| cellular response to hormone stimulus | 3 | 3.30E-02 | |
| cardiac muscle contraction | 3 | 3.30E-02 | |
| regulation of ion transmembrane transport | 4 | 3.30E-02 | |
| organ regeneration | 3 | 3.50E-02 | |
| SA node cell action potential | 2 | 3.70E-02 | |
| cellular protein metabolic process | 4 | 3.90E-02 | |
| cellular response to calcium ion | 3 | 4.10E-02 | |
| negative regulation of interleukin-1 beta secretion | 2 | 4.30E-02 | |
| regulation of ventricular cardiac muscle cell membrane depolarization | 2 | 4.30E-02 | |
| inhibitory postsynaptic potential | 2 | 4.90E-02 | |
| pattern recognition receptor signaling pathway | 2 | 4.90E-02 | |
| regulation of atrial cardiac muscle cell membrane depolarization | 2 | 4.90E-02 | |
| response to mechanical stimulus | 3 | 5.40E-02 | |
| lipoprotein biosynthetic process | 2 | 5.50E-02 | |
| atrial cardiac muscle cell action potential | 2 | 5.50E-02 | |
| retinoid metabolic process | 3 | 5.70E-02 | |
| positive regulation of lipid storage | 2 | 6.10E-02 | |
| dopamine biosynthetic process | 2 | 6.10E-02 | |
| positive regulation of transcription from RNA polymerase I promoter | 2 | 6.10E-02 | |
| blood vessel endothelial cell migration | 2 | 6.10E-02 | |
| sleep | 2 | 6.70E-02 | |
| transmembrane transport | 5 | 6.90E-02 | |
| transport | 6 | 6.90E-02 | |
| parturition | 2 | 7.30E-02 | |
| negative regulation of G-protein coupled receptor protein signaling pathway | 2 | 7.30E-02 | |
| maintenance of gastrointestinal epithelium | 2 | 7.30E-02 | |
| response to caffeine | 2 | 7.30E-02 | |
| sodium ion transmembrane transport | 3 | 7.80E-02 | |
| prepulse inhibition | 2 | 7.90E-02 | |
| membrane depolarization during cardiac muscle cell action potential | 2 | 7.90E-02 | |
| response to nutrient | 3 | 8.00E-02 | |
| response to endoplasmic reticulum stress | 3 | 8.10E-02 | |
| regulation of short-term neuronal synaptic plasticity | 2 | 8.50E-02 | |
| cellular glucose homeostasis | 2 | 8.50E-02 | |
| fatty acid oxidation | 2 | 8.50E-02 | |
| lipoprotein transport | 2 | 9.10E-02 | |
| glutamate receptor signaling pathway | 2 | 9.10E-02 | |
| ventricular cardiac muscle cell action potential | 2 | 9.10E-02 | |
| sperm capacitation | 2 | 9.60E-02 | |
| negative regulation of tumor necrosis factor-mediated signaling pathway | 2 | 9.60E-02 | |
| cardiac muscle cell action potential involved in contraction | 2 | 9.60E-02 | |
| proteolysis | 7 | 9.70E-02 | |
