# Supplementary figures and images for: AGR2 expression as a predictive biomarker for therapy response in esophageal squamous cell carcinoma
Source: PLoS One. 2022 Nov 3;17(11):e0276990. doi: 10.1371/journal.pone.0276990 (PMC9632826; doi:10.1371/journal.pone.0276990)

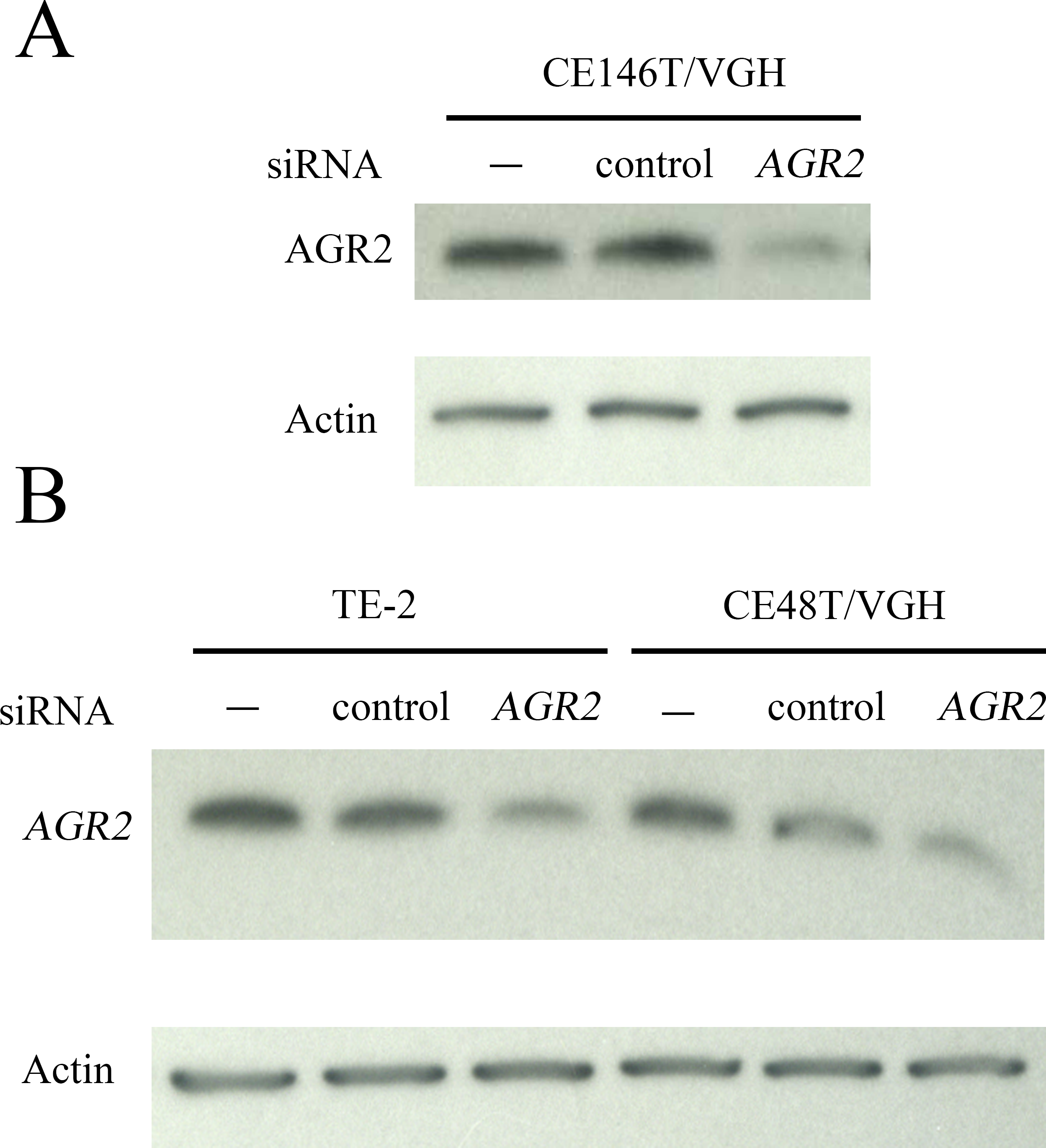

Supplement: S1 Fig — The amounts of 25μM control siRNA (si-Control) and 25μM AGR2 siRNA (si-AGR2) were transfected into esophageal cells. The loading control was β-actin expression level. (A) CE146T/VGH, (B) TE-2 and CE48T/VGH. (TIF) [file pone.0276990.s003.tif]

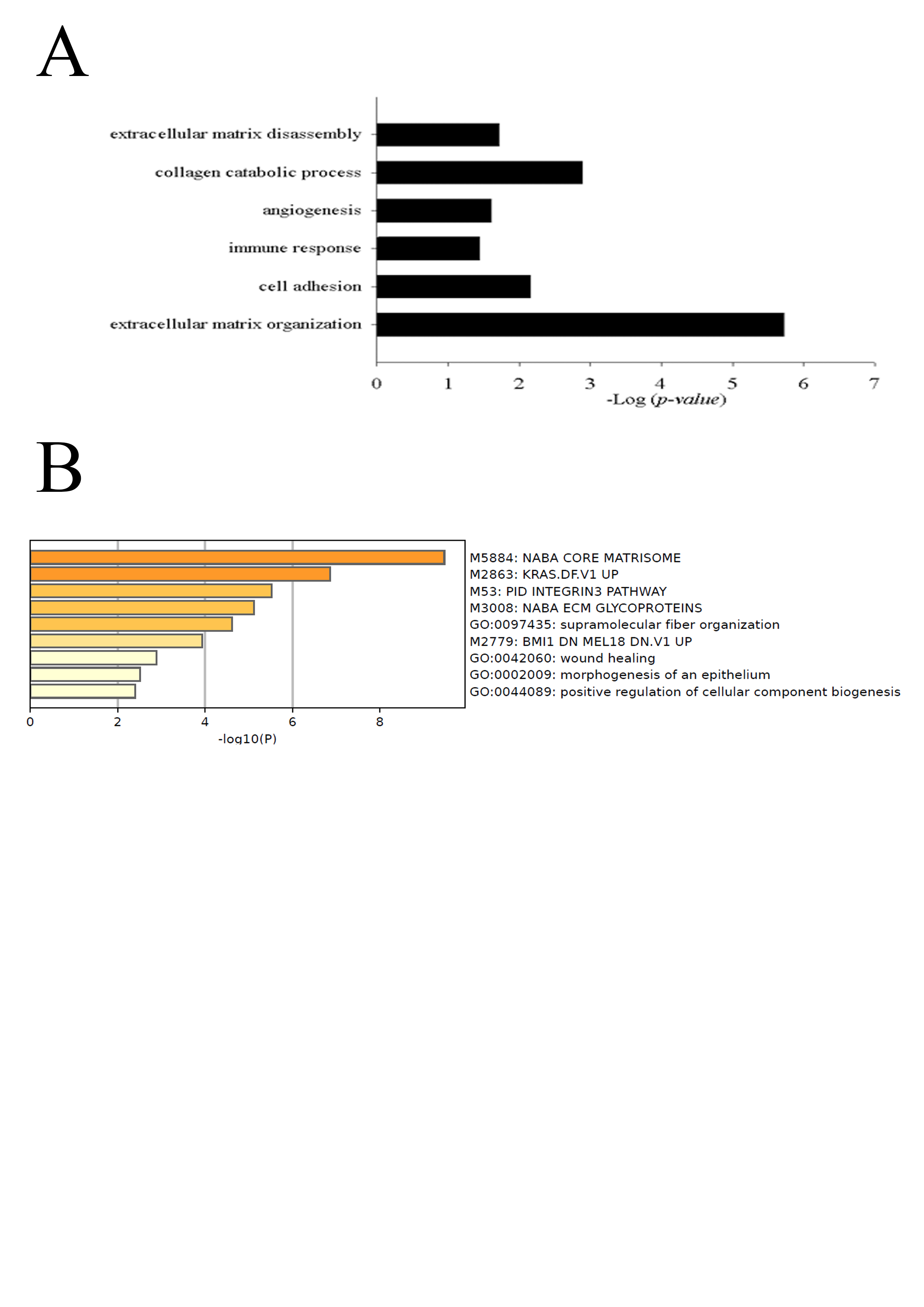

Supplement: S2 Fig — The bar graph showed the down-regulated gene expression pattern according to functional enrichment analysis from DAVID (A) and Metascape (B) online. (TIF) [file pone.0276990.s004.tif]
